# Molecular mechanisms of lipid sensing in human cholecystokinin-secreting enteroendocrine cells

**DOI:** 10.1016/j.molmet.2026.102448

**Published:** 2026-09-28

**Authors:** Marta Santos-Hernández, Mae Tabbada, Nademah Tamkin, Linxia Li, Nunzio Guccio, Adam Davison, Chris A. Smith, Richard Kay, Constanza Alcaino, Mirza Baig, Austin Punnoose, Yining Xu, Rula Bany Bakar, Frank Reimann, Fiona M. Gribble

**Affiliations:** University of Cambridge, Institute of Metabolic Science and MRC Metabolic Research Laboratories, Addenbrooke's Hospital, Cambridge, United Kingdom

**Keywords:** Cholecystokinin (CCK), Gut-brain-axis, Lipid-sensing, Hormone secretion

## Abstract

**Objectives:**

Cholecystokinin (CCK) has been implicated in coordinating lipid digestion and satiety. However, the molecular mechanisms underlying nutrient-dependent CCK-release from human I-cells are not well understood, at least in part due to the cells’ scattered distribution and a lack of reliable CCK-assays. Here we used human intestinal organoids to characterize lipid-sensing mechanisms underlying CCK release.

**Methods:**

Human duodenal organoids were genetically engineered using CRISPR-Cas9 to insert either the fluorescent protein Venus or the cAMP reporter Epac-S-H187 at the CCK locus. Transcriptomic profiling of CCK-positive and negative cells was performed following fluorescent-activated cell sorting. Intracellular calcium and cAMP as well as CCK-GRAB-sensor secretory responses were assessed during live-cell imaging, and CCK secretion was quantified by LC-MS/MS. To evaluate lipid-sensing pathways, *FFAR1*, *FFAR4*, and *GPR119* knockout organoids were generated.

**Results:**

Transcriptomic analysis identified expression of lipid-sensing GPCRs in I-cells, including *FFAR1*, *FFAR4*, *FFAR2*, *GPR119*, *GPBAR1*, *OR51E1* and *OR51E2*. Fatty acids and FFAR1 agonists increased intracellular calcium, whereas agonists of GPBAR1 and GPR119, and short-chain fatty acids elevated cAMP. CCK release was triggered by FFAR1, GPBAR and GPR119 agonists, and fatty acids with chain-length > C_8_. *FFAR1* knockout organoids exhibited impaired Ca^2+^ and CCK secretory responses to fatty acids, whereas responses of *FFAR4* KO organoids were like wild-type.

**Conclusions:**

In human I-cells, FFAR1 plays a crucial role in lipid-induced CCK release, whereas FFAR4 appears to be redundant in this context. These findings improve understanding of human CCK physiology and identify potential therapeutic targets for metabolic regulation and appetite control.

## Highlights

• Fluorescent tagging enabled first in-depth characterization of I cells in human organoids.
• Transcriptome and peptidome combined with live-cell imaging identifies I-cell sensors.
• FFAR1 mediated increase in cytosolic Ca^2+^ is critical for lipid stimulated CCK secretion.
• FFAR4 is dispensable for lipid stimulated CCK release from human I-cells.

## 1 Introduction

Cholecystokinin (CCK) is produced by a specialized subset of enteroendocrine cells (EECs), known as I-cells, which are located predominantly in the upper small intestine. CCK is best known for its role in coordinating nutrient digestion by stimulating gallbladder contraction, increasing pancreatic enzyme secretion, and delaying gastric emptying. Beyond its digestive functions, CCK also transmits signals of nutrient intake to the brain via vagal afferents, thereby suppressing further food intake but has also been implicated in learned preference for nutritious sweet and fatty foods [1,2]. Indeed, vagal neuronal responses to intestinal administration of sugar and fats were abolished when CCK signaling was blocked with a CCKAR antagonist, or when CCK-receptor expressing vagal afferent neurons in the nodose ganglion were ablated with CCK-saponin [3], suggesting this hormone as a key transmitter mediating sugar/nutrient-sensing in the gut–brain axis [4].

In humans, plasma CCK levels increase after the ingestion or intraduodenal administration of meals containing protein, fat, and to a lesser extent carbohydrate [[5], [6], [7], [8]]. Several studies reported that the administration of different length chain fatty acids robustly stimulated CCK secretion. For instance, intraduodenal infusion of soybean oil increased plasma CCK, whereas a mixture of medium-chain fatty acids (MCFAs), including caprylic (C_8_) and capric (C_10_) acids esterified with glycerol, had no significant effects [9]. Other studies revealed a greater CCK response following intraduodenal infusion of lauric (C_12_) than capric acid [10,11]. It was also reported that oral administration of olive oil increased plasma CCK, whereas 1,3-dioctanoyl-2-oleoyl glycerol (digested to C_8_ fatty acids and 2-mono-oleoylglycerol (2-OG)) did not elicit a significant response, highlighting the importance of long-chain fatty acids (LCFA) in CCK secretion [[10], [11], [12]].

Nutrient detection in EECs occurs either via membrane transporters or through the activation of G protein–coupled receptors (GPCRs) [13]. Activation of these pathways triggers intracellular signaling cascades, including elevation of calcium (Ca^2+^) and cyclic adenosine monophosphate (cAMP) levels, ultimately leading to hormone secretion. Despite the physiological importance of CCK, the cellular and molecular characterization of human CCK–secreting cells remains limited, largely due to the lack of reliable *in vitro* models, the difficulty of distinguishing I-cells from other intestinal epithelial cell populations and the cross-reactivity of most commercial CCK assays with gastrin. GPCR expression in EEC populations in murine and human intestinal organoids has been assessed previously by quantitative PCR and single-cell RNA sequencing [[14], [15], [16], [17], [18]], revealing that I-cells express a range of lipid-relevant nutrient-sensing receptors for fatty acids, bile acids, and monoacylglycerols. Functional studies in mice highlighted the importance of the long-chain fatty acid receptors Ffar1 and Ffar4, for CCK responses to olive oil [19] or corn oil [20]. However, similar data on the molecular mechanisms underlying lipid detection by human I-cells are lacking, yet are crucial for understanding postprandial physiology.

This study aimed to characterize the mechanisms underlying human CCK secretion, with a focus on lipid digestion products. Human CCK-secreting I-cells were genetically tagged with fluorescent markers, enabling their identification and purification for subsequent transcriptomic, peptidomic analysis as well as characterization of stimulus secretion coupling using live cell imaging and electrophysiological techniques. Knock-out of *FFAR1* confirmed a critical role of this receptor for lipid induced CCK-secretion, whereas *FFAR4* appeared to be redundant.

## 2 Materials and methods

### 2.1 Human organoid culture. Generation of CCK-Venus and CCK-Epac-S-H187 human duodenal organoids

Human duodenal organoids were generated from fully anonymized specimens provided by Addenbrooke's Hospital Tissue Bank under approval by the East of England–Cambridge Central Research Ethics Committee approval (ref: 09/H0308/24). Duodenal organoids were maintained as previously described [21].

CCK-Venus and CCK-Epac-S-H187 organoids were generated using CRISPR-associated protein 9 (Cas9) induced homology-directed repair (HDR) to knock in either Venus or the FRET cAMP reporter Epac-S-H187 [22] behind a picornavirus peptide-2A (P2A) sequence enabling bicistronic expression under the *CCK* promoter. Plasmids were produced using Gibson cloning (New England Biolabs). Primers designed to generate the CCK-Venus and CCK-Epac-S-H187 plasmids, along with guide RNAs and screening primers, are provided in Table S1 and Table S2. Guides targeting exon 5 of the *CCK* locus immediately prior to the stop codon (Table S1; Figure 1A). were cloned into plasmids co-expressing nuclear-targeted Cas9 as previously described [23,24]. Organoids were digested into single cells and electroporated with the single guide RNA (sgRNA)-Cas9 and the donor plasmid, as described previously [24]. To establish the organoid lines, 0.5 mg/mL G418 was added to the media 4–6 days post-electroporation and surviving organoids were manually picked and seeded individually. DNA was extracted using QuickExtract DNA Extraction Solution (Lucigen Corporation, USA), and correct integration screened by PCR and confirmed by Sanger sequencing (Source BioScience). To promote I-cell differentiation, EGF was removed from the media and 10 μmol/l Notch inhibitor DAPT (Generon, Slough, UK) and 100 nmol/l MEK inhibitor PD0325901 (Merck, Gillingham, UK) were transiently supplemented into the media overnight. Subsequently, organoids were cultured in standard media without EGF until optimal I-cell differentiation, usually observed around 5–7 days later.

**Figure 1:**
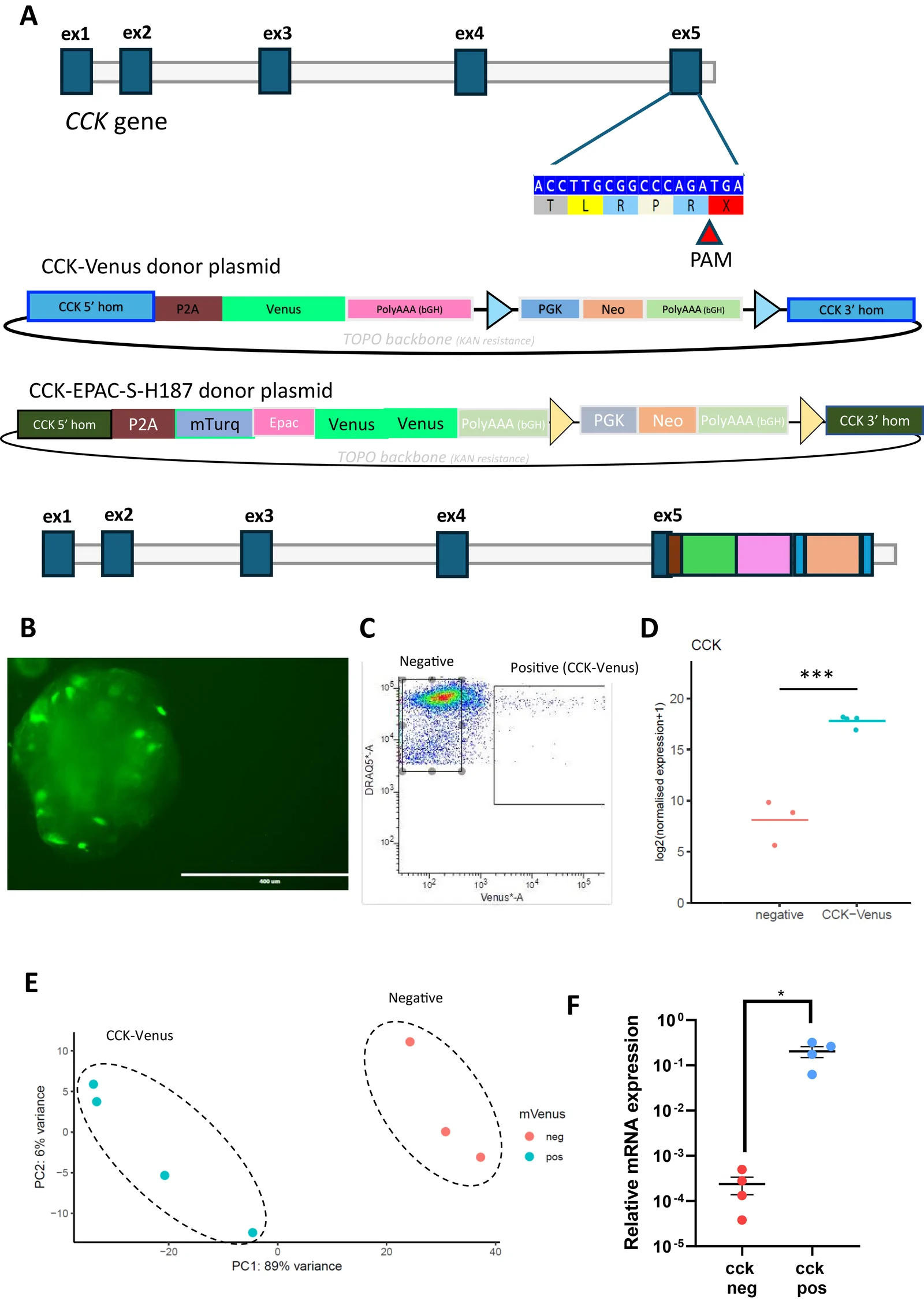
Generation of CCK-Venus and CCK-Epac-S-H187 human duodenal organoids. (A) Schematic showing the insertion of Venus or Epac1-S-H187 sensor in exon 5 of the CCK gene by CRISPR-Cas9 homology-directed repair. (B) Representative image of CCK-Venus human duodenal organoids. (C) Representative fluorescent-activated cell sort (FACs) plot of 500 k events. (D) Differential CCK expression in Venus positive (blue, n = 4) and negative (red, n = 3) cell population. Data are presented as mean ± SEM; ∗∗∗p < 0.001 two-tailed t test. (E) Principal component analysis comparing CCK-Venus positive (blue) and negative (red) cell populations following bulk RNA sequencing. (F) Expression of CCK mRNA in Venus-negative (red) and Venus-positive (blue) sorted cells by qPCR, expressed relative to β-actin. Data are presented as mean ± SEM; ∗p < 0.05 non-parametric Mann–Whitney test.

### 2.2 Generation of FFAR1, FFAR4 and GPR119 knock-out (KO) in CCK-Venus human duodenal organoid lines

*FFAR1*, *FFAR4* and *GPR119* KO organoids were generated by CRIPSR-Cas9 non-homologous end joining (NHEJ). Guides (FFAR1: targeting transmembrane domains 2 and intracellular loop 3 (exon 1); GPR119: targeting transmembrane domain 2 and 3 (exon 1)) were purified as described previously [25]. FFAR4 guides were designed to target extracellular loop 1 and transmembrane domain 3 (exon 1). Guide sequences (see Table S1) were cloned into plasmids for electroporation as above. Genomic DNA was extracted and screened by PCR amplification (primer sequences provided in Table S2) to identify clones with biallelic deletions.

### 2.3 Fluorescence-activated cell sorting (FACS)

Cell sorting was performed as described previously [23]. Briefly, differentiated organoids were digested into single cells with TryPLE prior to resuspension in Hanks’ Balanced Salt Solution (without Ca^2+^ or Mg^2+^) supplemented with 10% fetal bovine serum and 10 μM Rho-kinase (ROCK) inhibitor Y-27632. Live DAPI-negative, DRAQ5-positive single cells were sorted based on Venus fluorescence using a FACS Melody cell sorter (BD Biosciences). Venus positive and negative cells were collected in 350 μL RLT buffer (Qiagen) supplemented with 1% β-mercaptoethanol for RNA extraction, or 350 μL 6 M guanidine hydrochloride for peptidomics.

### 2.4 Quantitative PCR (qPCR) and bulk RNA sequencing

RNA was extracted using RNAeasy Micro Plus kit (Qiagen) and quantified using RNA 6000 Pico Kit and Bioanalyser 2000 (Agilent). cDNA libraries were generated as previously described [21,23]. Differential expression comparing CCK Venus positive vs negative cell extracts was calculated using the Wald test (default in DESeq2, version 1.42.0 [26]). The raw sequences and unfiltered counts matrix for the bulk RNA sequencing are available at the Gene Expression Omnibus (GEO) under accession number GSE341673.

RT-qPCR was performed on complementary DNA (cDNA) prepared with Super Script IV Reserve Transcriptase (Invitrogen), using the following Taq Man probes: ACTB, Hs01060665_g1; CCK, Hs00174937_m1.

### 2.5 Peptidomic analysis of sorted cells

Sorted cells in 6 M guanidine hydrochloride (GuHCl) were subjected to three freeze-thaw events. Cell lysates in GuHCl (350 μL) were supplemented with 50 μL of 5 ng/mL bovine insulin, resulting in a final insulin internal standard concentration of 0.625 ng/mL. A further 500 μL of 0.1% formic acid was added to dilute the GuHCl prior to SPE extraction. Samples were loaded onto a HLB PRiME μ-Elution solid-phase extraction plate (Waters) and washed with 200 μL 0.1% formic acid in water and 200 μL 5% methanol with 1% acetic acid in water. Peptides were eluted into a clean Eppendorf LoBind plate with two 30 μL aliquots of 60% methanol in 10% acetic acid in water. Samples were evaporated under oxygen free nitrogen and reconstituted into 75 μL of 10 mM DTT in 50 mM ammonium bicarbonate and reduced for 60 min at 60 °C. Alkylation was performed by adding 20 μL of 100 mM iodoacetamide in 50 mM ammonium bicarbonate and leaving in the dark for 30 min. Alkylation was quenched by light-exposure for 20 min. 20 μL of 1% TFA was added prior to LC-MS/MS analysis on a Water M-Class nano-LC system coupled to a SCIEX 7600 ZenoToF mass spectrometer. LC-MS/MS files were searched against the Human Uniprot database (accessed, August 2025) using PEAKS (v12.5, BSI). Search parameters included a no-enzyme setting, precursor (25 ppm) and product ion (0.05 Da) tolerances, a fixed modification of carbamidomethylation on cysteine residues, and variable modifications of methionine oxidation, N-terminal pyroglutamate, N-terminal acetylation, and C-terminal amidation. The data were filtered to include only protein identifications with a <1% false discovery rate (FDR) and at least one unique peptide. The sorted cell peptidomics data have been deposited to the ProteomeXchange Consortium via the PRIDE partner repository with the data identifier PXD075708.

### 2.6 Generation of 2D monolayer cultures

CCK-Venus or CCK-Epac-S-H187 organoids were digested and seeded in two-dimensional (2D) monolayer cultures. Cells were seeded onto 4% Matrigel (Corning) pre-coated 24-well plates (secretion assays), or 35 mm glass-bottom dishes (MatTek: for GRAB, calcium and cAMP imaging), or 35 mm plastic dishes (electrophysiology) and incubated at 37 °C (5% CO_2_) for 18–24 h [21,24].

### 2.7 Secretion assays

The cultures were washed twice with saline buffer (138 mM NaCl, 4.5 mM KCl, 4.2 mM NaHCO_3_, 1.2 mM NaH_2_PO_4_, 2.6 mM CaCl_2_, 1.2 mM MgCl_2_, 10 mM HEPES,1 mM glucose; adjusted to pH 7.4 with NaOH) supplemented with fatty acid-free bovine serum albumin (BSA, 0.01%) following 30 min incubation with the same buffer prior the experiments. Test reagents were dissolved in the buffer (250 μL per well) and incubated with the 2D culture monolayers at 37 °C for 2 h. All secretion plates included vehicle-treated samples as the negative control (basal condition) and 10 μM forskolin plus 100 μM IBMX in 138 buffer containing 10 mM glucose as the positive control (10G + F/I). The supernatants were then transferred into Lobind tubes (Eppendorf), centrifuged at 1000 g for 2 min at 4 °C, and 200 μL of the resulting supernatants were snap-frozen. The agonists and their concentrations used for the secretion and live-cell imaging experiments are listed in Table 1.

**Table 1:** Agonists used in the secretion and live-cell imaging experiments. The table lists each reagent, its supplier, the catalogue number, and the concentration(s) used in the study.

Reagent		Supplier	Catalogue number	Concentration tested
AM-1638	FFAR1 agonist	Eli lilly & co. (Gift) Generon	HY-13467	10 μM
AR231453	GPR119 agonist	Sigma–aldrich	SML1883	100 nM
Forskolin	Adenylate cyclase activator	Sigma–aldrich	F6886	10 μM
GPBAR-A	GPBAR1 agonist	Sigma–aldrich	SML1207	3 μM
IBMX (3-isobutyl-1-methylxanthine)	Phosphodiesterase inhibitor	Sigma–aldrich	I7018	100 μM
Isovaleric acid	OR51E1 agonist	Acros organics	156,690,025	1 mM
Sodium acetate trihydrate	FFAR2 agonist	Sigma–aldrich	6131-90-4	1 mM
Sodium butyrate	FFAR2 agonist	Sigma–aldrich	B5887	1 mM
Sodium propionate	FFAR2 agonist	Sigma–aldrich	P1880	1 mM
TUG891	FFAR1/4 agonist	Cayman chemical	17,035	30 uM and 1 uM
Sodium octanoate	FFAR1/4 agonist	Sigma–aldrich	C5038	1 mM
Sodium decanoate	FFAR1/4 agonist	Sigma–aldrich	C4151	1 mM
Dodecanoate	FFAR1/4 agonist	Sigma–aldrich	L9755	200 uM
Sodium oleate	FFAR1/4 agonist	Sigma–aldrich	O7501	200 uM
Eicosapentaenoic acid sodium salt	FFAR1/4 agonist	Sigma–aldrich	E6627	200 uM

Abbreviations: free fatty acid receptor 1–4 (FFAR1-4), olfactory receptor 51E1 (OR51E1), G protein-coupled bile acid receptor 1 (GPBAR1), G protein-coupled receptor 119 (GPR119).

A calibration line of preproCCK21-44 over a range of 1–1000 pg/mL was generated in 0.01% BSA in water in Eppendorf LoBind tubes. A total of 200 μL of each standard was transferred to a plate and 50 μL of 750 pg/mL of a stable isotope-labeled ppCCK21-44 (Biosynth) in 1% formic acid was added to each sample. Supernatant samples had 50 μL of internal standard added prior to thawing on ice and were then transferred to the 96 well plate and vortexed to mix. Samples were transferred to a Waters HLB PRiME μ-Elution SPE plate as described above. After elution, samples were diluted with 75 μL of 0.1% formic acid in water prior to targeted LC-MS/MS analysis. Samples were injected onto an M Class Acquity (Waters) microflow LC system coupled to a TQ-XS triple quadrupole mass spectrometer with an ionKey interface (Waters). The sample (25 μL) was injected onto a nanoEase M/Z Peptide BEH C18 Trap Column (130 Å, 5 mm, 300 mm 50 mm, Waters) at 12.5 μL/min for a 5-min load, with mobile phases set to 95% A (0.1% formic acid in water) and 5% B (0.1% formic acid in acetonitrile). The iKey HSS T3 Separation Device (100 Å, 1.8 mm, 50 mm, Waters) was set at 45 °C and the analytes were separated over a 19-min gradient from 5% to 45%B, at the flow rate of 3 μL/min. The iKey was flushed for 2.5 min at 85% B before returning to initial conditions, resulting in an overall run time of 35 min. The selected reaction monitoring (SRM) transitions used for ppCCK21-44 were 842.10/612.99 and 842.1/918.99, whilst 846.39/616.94 and 846.39/926.0 were used for the stable isotope labelled peptide, all with a dwell time of 20 ms. Data were processed on MassLynx (v 4.2, Waters).

### 2.8 Calcium imaging

Live single-cell calcium imaging of Venus-expressing I-cells was conducted following loading with Fura-2AM (5 μmol/l) (Thermo Fisher Scientific, Bishops Stortford, UK). Cells were mounted on an inverted microscope (Olympus IX71) and imaged at 2s intervals during continuous perfusion with saline buffer supplemented with 0.1 mM glucose and 0.1% BSA in the presence or absence of test stimuli (Table 1). Fura-2 fluorescence ratios (340/380 nm) were calculated after background subtraction using MetaFluor software (Molecular Devices). Responses are expressed as the fold change in Fura-2 ratio fluorescence in the test solution (maximum value recorded during stimulus perfusion) relative to baseline (maximum value recorded during saline buffer perfusion during the 1 min preceding stimulus onset).

### 2.9 cAMP imaging

cAMP-dependent FRET imaging was performed on differentiated CCK-Epac-S-H187 organoids. Live cells were imaged using an inverted microscope (Olympus IX71), as outlined above. Intracellular cAMP levels were recorded at 5s intervals during continuous perfusion with saline buffer in the presence or absence of test reagents (Table 1), using MetaFluor software (Molecular Devices, UK). Cells were excited at 435 nm, and YFP and CFP emissions were collected via an OptoSplit device (470/24 nm and 535/30 nm emission filters, respectively; Cairn Research, Faversham, UK) prior to detection with an Orca-ER camera (Hamamatsu, Welwyn Garden City, UK). CFP/YFP ratios were calculated following background subtraction. Responses are expressed as the fold change between the maximum CFP/YFP ratio recorded during stimulus perfusion and the maximum ratio recorded during saline buffer perfusion in the 1 min preceding stimulus onset.

### 2.10 GRAB sensor. Generation and co-culture

The CCK1 GRAB sensor plasmid (pDisplay-GRAB_ CCK1.0-IRES-mCherry, addgene plasmid #208672) was kindly provided by the laboratory of Yulong Li [27]. The CCK1 GRAB green sensor plasmid, co-expressing mCherry, was transfected into HEK293 cells using TransIT-293 (Mirus Bio) according to the manufacturer's instructions. HEK293 cells expressing the GRAB sensor (24 h after transfection) were tested for their responsiveness to oleic acid and CCK and only CCK application evoked a signal. GRAB sensor expressing HEK293 cells (24 h after transfection) were subsequently co-cultured with cultures from WT or *FFAR1* KO I-cell labelled organoids. Dissociated CCK-Venus organoids were mixed with HEK293 cells at an approximate 2:1 ratio and seeded onto 4% Matrigel or 35 mm glass-bottom dishes. HEK293 cells and CCK-Venus organoids were imaged using an inverted confocal microscope (Olympus IX71) equipped with a 40 × oil-immersion objective as described above. Image acquisition was performed using MetaFluor software (Molecular Devices). Green fluorescence was excited at 490 nm and detected using a 525/50-nm emission filter, while red fluorescence was excited at 550 nm and detected using a 595/50-nm emission filter. Responses are expressed as the fold change in green fluorescence in the test solution (maximum value recorded during stimulus perfusion) relative to baseline (maximum value recorded during saline buffer perfusion during the 1 min preceding stimulus onset).

### 2.11 Electrophysiology

Human CCK-Venus duodenal organoids were plated as a 2D culture 24 h prior to recording, as described above. Perforated current clamp recordings were performed using a HEKA EPC-10 amplifier and Patchmaster software (HEKA). During recordings, cells were continuously perfused with an extracellular solution consisting of (mmol/l) 138 NaCl, 4.5 KCl, 10 HEPES, 4.2 NaHCO_3_, 2.6 CaCl_2_, 1.2 MgCl_2_, 1.2 Na_2_HPO_4_, 1 d-Glucose. Patch pipettes were pulled from borosilicate glass (1.5 mm OD, 1.17 mm ID, Harvard Apparatus) using a PC-100 puller (Narishige) and had resistances of 2.5–3.5 MΩ when filled with pipette solution. Pipettes were filled with an intracellular solution consisting of (mmol/l): 76 K_2_SO_4_, 10 NaCl, 10 KCl, 10 HEPES, 55 sucrose, 1 MgCl_2_; adjusted to pH 7.2 with KOH. Amphotericin-B was added to the intracellular solution on the day of recording to a concentration of 200 μg/mL.

To measure action potential firing, current was injected to maintain the cells at a holding potential of −60mV and 10 sweeps of square current steps of increasing amplitude (2000 ms, Δ1-4 pA) were applied. Analysis of action potential peak, half-width and threshold were performed using RStudio (Posit).

### 2.12 Data analysis

Data are presented as mean ± SEM throughout, with individual data points shown on graphs where appropriate. Statistical analyses were conducted using GraphPad Prism (version 8) or DESeq2 for RNA sequencing data. Differences between groups were assessed using paired or unpaired t-tests, one- or two-way analysis of variance (ANOVA), or the Kruskal–Wallis test, as indicated in the figure legends. For calcium and cAMP imaging experiments, cells were included in the analysis only if they exhibited a response to the positive control (KCl for calcium imaging or forskolin/IBMX for cAMP imaging) corresponding to at least a 10% increase. Cells were classified as “responders” to a given test agent when the z-score exceeded 3 for at least two consecutive time points during perfusion of the test compound. The z-score was calculated as: z = (F_t_ − mean F_b_)/SD_F_b_, where F_t_ represents the fura-2 or FRET ratio at time point during application of the stimuli, and F_b_ represents the ratio under basal conditions. The mean F_b_ and SD_F_b_ were derived from the 60 s of data collected immediately prior to addition of the test agent. To determine if a given stimulus elicited a statistically significant calcium or cAMP response across all tested cells (including those classified as non-responders), Fura-2 or FRET ratios measured during application of the test agent were normalized to the basal ratio for each individual cell. Significant increases above baseline were evaluated using a one-sample Wilcoxon test against a baseline value of 1.

## 3 Results

### 3.1 Generation of CCK-Venus and CCK-Epac-S-H187 human duodenal reporter lines

To selectively label human I-cells, human CCK-Venus intestinal organoids were generated by CRISPR-Cas9-mediated HDR. This involved inserting a P2A sequence followed by the Venus coding sequence immediately after the CCK coding region in exon 5 (Fig. 1A). A similar approach was used to generate a CCK–Epac-S-H187 reporter line (Fig. 1A). In mature organoids, sporadic yellow fluorescent cells were observed, displaying a distribution pattern characteristic of EECs (Fig. 1B). After dissociation, CCK–Venus organoids were fluorescence-activated cell sorted (FACS) to isolate Venus-positive and Venus-negative populations (Fig. 1C), which were analysed by bulk RNA sequencing. Principal component analysis (PCA) demonstrated clear separation between fluorescent and non-fluorescent cell populations (Fig. 1E). The RNA-seq data revealed strong enrichment (approximately 600-fold) of *CCK* transcripts in the fluorescent population compared to negative cells (Figure 1D,E), confirming that Venus-positive cells correspond to *CCK*-expressing I cells. qPCR analysis similarly showed a substantial enrichment of *CCK* mRNA levels in Venus-positive cells (approximately 800-fold; Fig. 1F).

### 3.2 Transcriptomic and peptidomic analysis of human I-cells

Venus-positive I-cells exhibited enriched expression of *CCK* as well as other hormones such as peptide YY (*PYY*), glucose-dependent insulinotropic polypeptide (*GIP*), proglucagon (*GCG*), neurotensin (*NTS*), urocortin 3 (*UCN3*) and gastrin (*GAST*) by bulk RNA sequencing (Figure 2A,D). Ghrelin (*GHRL*) and secretin (*SCT*) were also detected at lower levels, along with somatostatin (*SST*), although SST expression was lower than in the Venus-negative population (Figure 2A,D). Motilin (*MLN*) was also expressed in Venus positive and negative cells (Fig 2D). LC-MS/MS peptidomic analysis of isolated Venus-positive I-cells confirmed production of several gut hormone peptides besides CCK, including PYY, GAST, GIP and MLN (Fig 2E).

**Figure 2:**
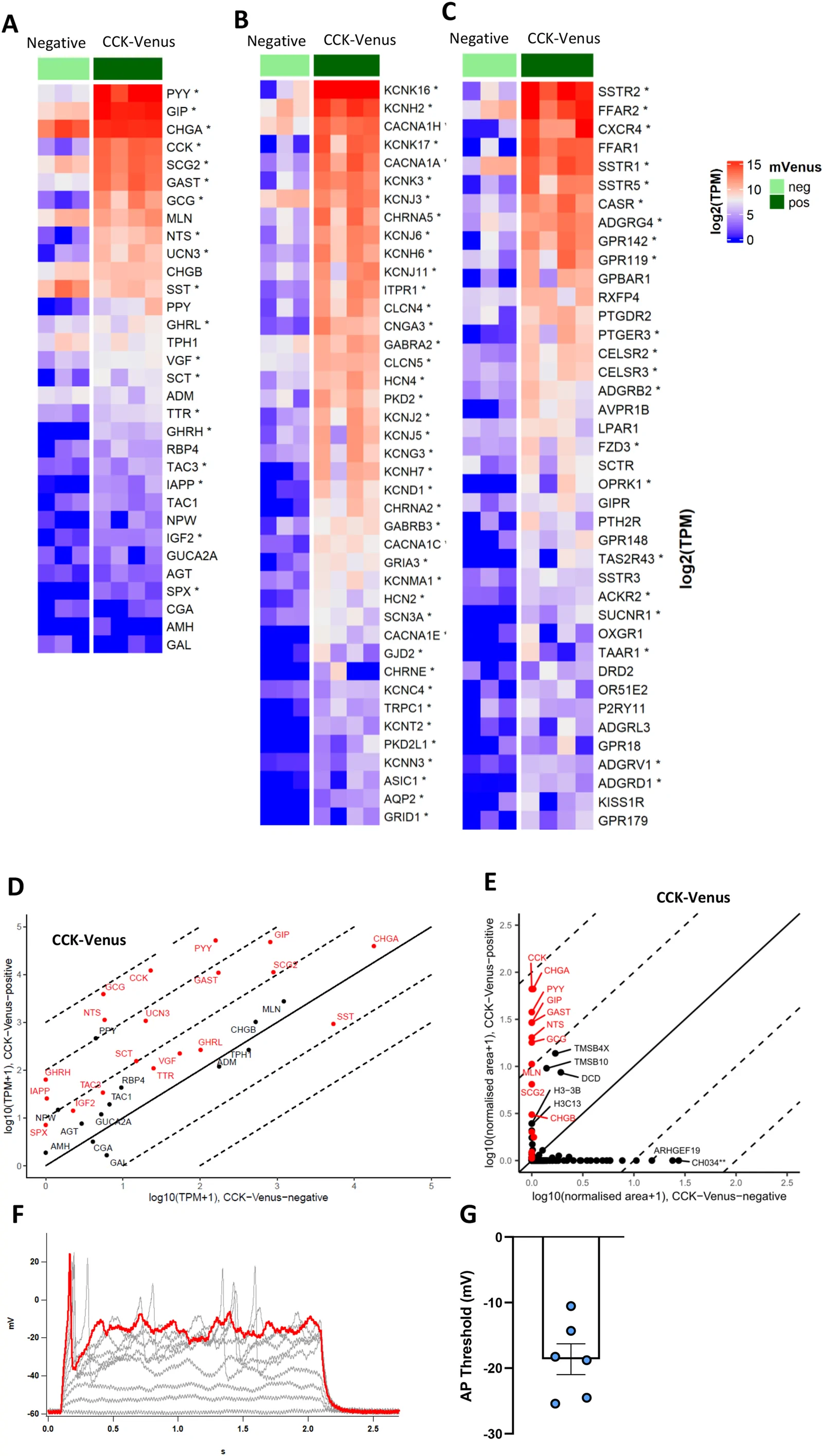
Transcriptomics and peptidomic analysis, and electrical activity of human I cells. (A) Heatmap showing expression of the top 30 highest expressed hormones and secretory proteins in CCK-Venus compared to negative cells. (B) Heatmap showing expression of the top 40 highest ion channel and transporter genes enriched in CCK-Venus cells. (C) Heatmap showing expression of the top 40 highest GPCRs genes enriched in CCK-Venus cells. (A–C) ∗Significant differential expression between CCK-Venus I cells compared with the Venus-negative. (D) Transcriptomic analysis log10 plot showing expresion of genes of interest in CCK-Venus-positive (y-axis) Vs negative (x-axis), where significantly enriched genes (p < 0.05) are labelled in red. (E) LC-MS/MS peptidomic analysis of purified CCK-Venus positive and negative cells (the individual peptides detected are combined and associated to the parental protein, labeled by protein name and expressed as mean peak area) where significantly enriched proteins (p < 0.05) are labelled in red. (F) Example trace of a perforated current clamp recording of a human I-cell, with evoked action potentials elicited in response to increasing (Δ2pA) current injection. (G) Scatter plots showing action potential (AP) threshold potentials observed in different cells.

The RNA-seq data showed differential I-cell expression of voltage-gated ion channels previously identified in other electrically excitable EECs important for action potential generation and vesicular exocytosis (Fig 2B), including P/Q-type Cav2.1 (*CACNA1A*), L-type Cav1.2 (*CACNA1C*), and inward-rectifying channels Kir3.2/3.4/6.2 (*KCNJ3/6/11*). *CACNA1H* (T-type) was also highly expressed in I-cells, as previously found in human GIP secreting cells [21]. Expression of the K^+^ channel subunits *KCNK16*, *KCNH2* and *KCNK17* were also enriched. The voltage-gated Na ^+^ channel subunit *SCN3A* (Na_v_1.3) was detected but not enriched. Perforated patch current-clamp recordings of human organoid-derived I-cells were performed in 2D cultures, where increasing current injection was applied to evoke action potentials. We found that all (6/6) I-cells exhibited evoked action potentials in response to current injection, with a (mean ± SEM) action potential threshold of −18.6 +/−2.34 mV, action potential peak of +17.1 ± 1.5 mV, and action potential half-width of +32.3 ± 3.5 ms (Figure 1F,G), in line with findings from other enteroendocrine cells [21,24,28].

I-cells exhibited differential expression of a number of GPCRs previously implicated in EEC nutrient sensing (Fig. 2C), including *FFAR2*, *FFAR1* and *GPR119*, implicated in sensing of short-chain fatty acid (SCFAs), LCFAs and monoacylglycerols, respectively; *CASR* and *GPR142*, involved in amino acid (AA) sensing; and *GPBAR1*, which mediates bile acid sensing. *FFAR4*, which has been implicated previously in LCFA sensing, was expressed in both Venus positive and negative populations. Several GPCRs involved in hormone sensing, including somatostatin (*SSTR1/2/5*), secretin (*SCTR*) and GIP (GIPR) were expressed in the I-cell population, along with the kappa opioid receptor *OPRK1*. Significantly enriched in the I-cell population was the bitter-taste receptor *TAS2R43*, and *TAS2R31* was also expressed but not enriched. Interestingly, we found enrichment of some receptors involved in inflammation and immune responses, such as the chemokine receptor *CXCR4*, the succinate receptor *SUCNR1* and the prostaglandin receptors *PTGDR2* and *PTGER3*, involved in immune response modulation and gastrointestinal motility [29,30].

### 3.3 Stimulation of CCK secretion by GPCR agonists

CCK secretion experiments were performed by measuring preproCCK21-44 (ppCCK21-44) using LC-MS/MS, as described previously [31], in 2D cultured organoids (Figure 3A) to explore the function of the metabolite-sensing receptors FFAR1, FFAR2, FFAR4, GPR119 and GPBAR1. The synthetic and specific FFAR1 ligand, AM1638 at 10 μM, triggered a significant increase in CCK secretion (2.2 ± 0.3 fold) (Fig 3B). AM1638 promoted intracellular calcium responses in I-cells (Figure 3C,F), reflecting the reported Gq-coupling of this receptor. Since *FFAR4* is also expressed in Venus positive and negative cells, although not enriched, we tested the FFAR4/FFAR1 agonist TUG-891 in live-cell calcium imaging. This agonist has been claimed to be FFAR4 specific at lower concentrations (1 μM), whereas at higher concentrations (30 μM) can also target FFAR1 [32]. TUG-891 at 1 μM did not trigger a significant Ca^2+^ response, whereas at 30 μM it significantly increased intracellular calcium levels in I-cells (Figure 3D,F). By z-score analysis, AM1638 elicited significant Ca^2+^ responses in 18/24 I-cells, and TUG-891 at 30 μM triggered responses in 26/27 I-cells (Fig 3F).

**Figure 3:**
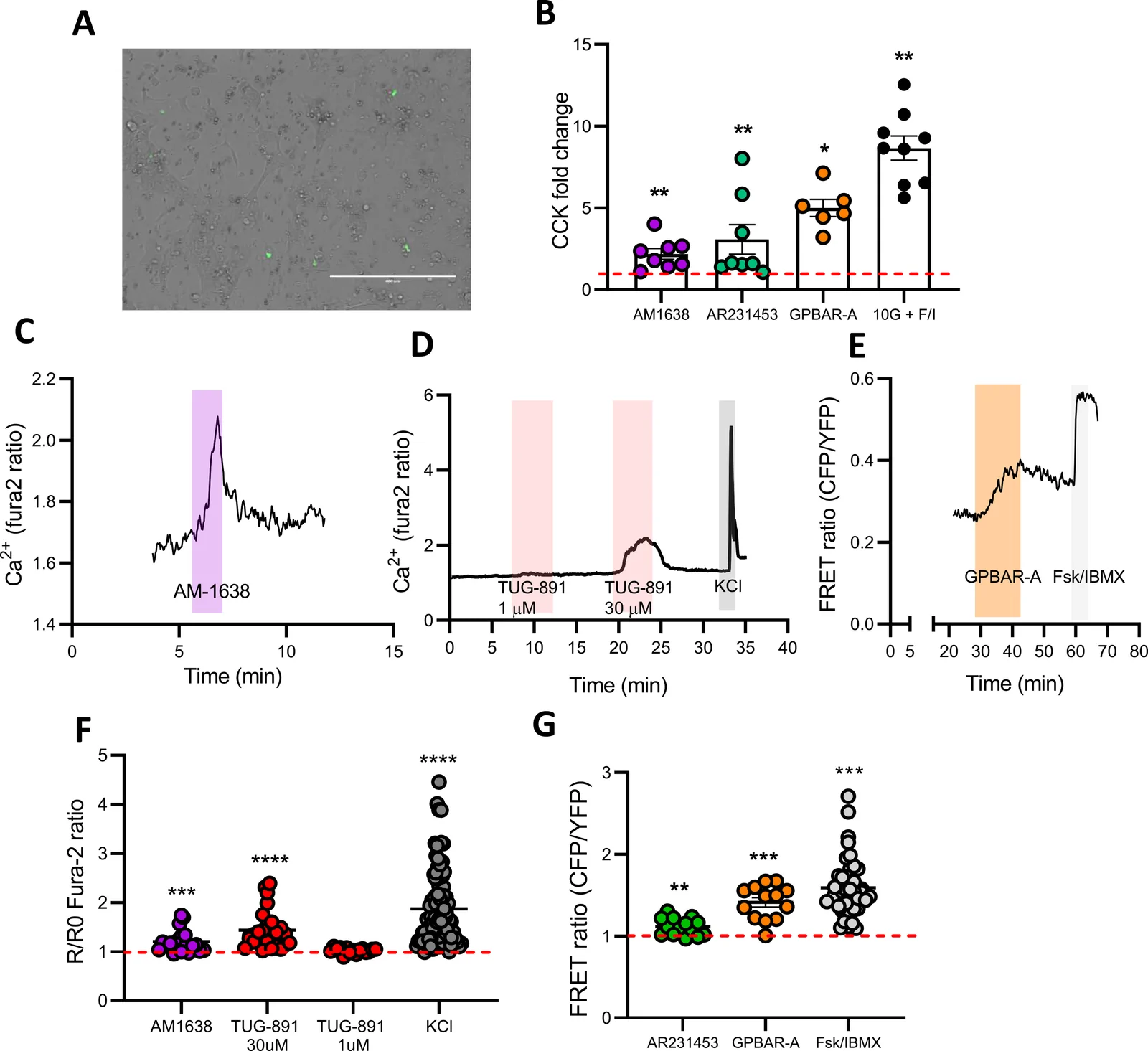
Stimulation of CCK secretion by GPCRs agonists. (A) Representative image of 2D-cultured CCK-Venus duodenal organoids used in secretion experiments. Green fluorescent cells correspond to human I cells. (B) Secretion of CCK from CCK-Venus human duodenal organoids in response to the stimuli indicated, expressed as fold change vs basal condition (1 mM glucose and 0.01% bovine serum albumin (BSA)) measured in parallel. The stimuli included AM1638 (10 μM), GPBAR-A (3 μM) and AR231453 (100 nM). All test solutions contained 1 mM glucose and 0.01% BSA (n = 6 wells from 3 independent experiments). Vehicle (basal condition) was used as the negative control and 10 μM forskolin plus 100 μM IBMX (138 buffer, 10 mM glucose (10G + F/I)) as the positive control. (C, D) Representative Fura-2 (340/380) ratio traces of single I cells perfused with (C) AM1638 (10 μM) and (D) TUG-891 (1 and 30 μM) and positive control KCl (50 mM). (E) Representative FRET (CFP/YFP) ratio trace of single I cell perfused with GPBAR-A (3 μM) and positive control forskolin (Fsk)/IBMX (10 μM/100 μM). (F) Mean data collected as in (C, D), shown as ratio between R (Fura-2 ratio during perfusion of stimulus) and R0 (Fura-2 ratio during perfusion of basal solution) (n = 11–27 cells from 3 to 6 independent experiments). (G) Mean data collected as in (E), shown as ratio between maximal CFP/YFP ratio (R) during perfusion of stimulus and maximal CFP/YFP ratio (R0) during perfusion of basal solution (n = 17–19 from 4 to 5 independent experiments). Data are presented as mean ± SEM. ∗p < 0.05, ∗∗p < 0.01, ∗∗∗p < 0.001, ∗∗∗∗p < 0.0001 b y one-sample Wilcoxon test (b, f, g).

Human FFAR4 exists as two splice isoforms, with the long variant, which contains a 16-amino acid insertion in the third intracellular loop, exhibiting weak Gi activity and no detectable Gs or Gq signalling in response to EPA, TUG-891, or linoleic acid (LA) [33]. To determine which isoform is expressed in our model, we performed PCR on cDNA generated from RNA isolated from CCK-Venus organoids. We found that human duodenal organoids predominantly express the long FFAR4 transcript (Fig. S2), which may explain the absence of an intracellular Ca^2+^ response following perfusion with 1 μM TUG-891.

Compared to FFAR1 agonist, the GPBAR1 agonist GPBAR-A (3 μM) and GPR119 agonist AR231453 (100 nM) appeared to have more marked effects on CCK secretion, inducing 5.0 ± 0.5 and 3.1 ± 0.9-fold increases, respectively (Fig 3B). As GPBAR1 and GPR119 are Gs-coupled, intracellular cAMP levels in I-cells were monitored using the CCK-Epac-S-H187 line. Consistent with the detection of mRNA for these receptors, GPBAR-A (3 μM) and AR231453 (100 nM) induced modest but significant cAMP responses in CCK-Epac-S-H187 cells (Figure 3E,G). By z-score analysis, GPBAR-A provoked a significant increase in the CFP/YFP ratio, a measure of intracellular cAMP, in 12/13 CCK-Epac-S-H187 cells, and responses to AR231453 were observed in 5/15 cells.

### 3.4 Free-fatty acid sensing in human I-cells

As described above, I-cells expressed multiple GPCRs capable of sensing fatty acids of different chain lengths, including *FFAR2* for SCFAs, *FFAR1* and *FFAR4* for MCFAs and LCFAs. They also expressed lower levels of the olfactory receptors *OR51E1* and *OR51E2* that can sense different length chain fatty acids [13]. As shown above, FFAR1 is known to be Gq-coupled promoting intracellular calcium responses in I-cells. Whilst FFAR4 has also been linked to Gq-coupled signalling, some studies have suggested a greater importance of β-arrestin–dependent signaling, with pathway activation influenced by the agonist and cellular context [34]. Hence, different chain-length fatty acids were tested for their ability to induce intracellular Ca^2+^. The LCFAs oleic acid (OA, 200 μM) and eicosapentanoic acid (EPA, 200 μM) elicited significant Ca^2+^ responses in 19/21 and 16/16 I-cells, respectively (Figures 4B and 6A). Dodecanoic acid (C_12_, 200 μM), decanoic acid (C_10_, 1 mM), and octanoic acid (C_8_, 1 mM) triggered moderate but significant Ca^2+^ responses in 19/29, 13/15 and 6/6 cells, respectively (Figure 4A,B, c). Regarding SCFAs, except for isovalerate (Isov), which did not elicit a significant increase in intracellular Ca^2+^, the other SCFAs tested (acetate (C_2_), propionate (C_3_) and butyrate (C_4_), all at 1 mM) induced significant Ca^2+^ responses in 4/10, 4/5 and 9/19 I-cells, respectively, consistent with the Gi/q coupling of FFAR2 (Figure 4A,B, c).

**Figure 4:**
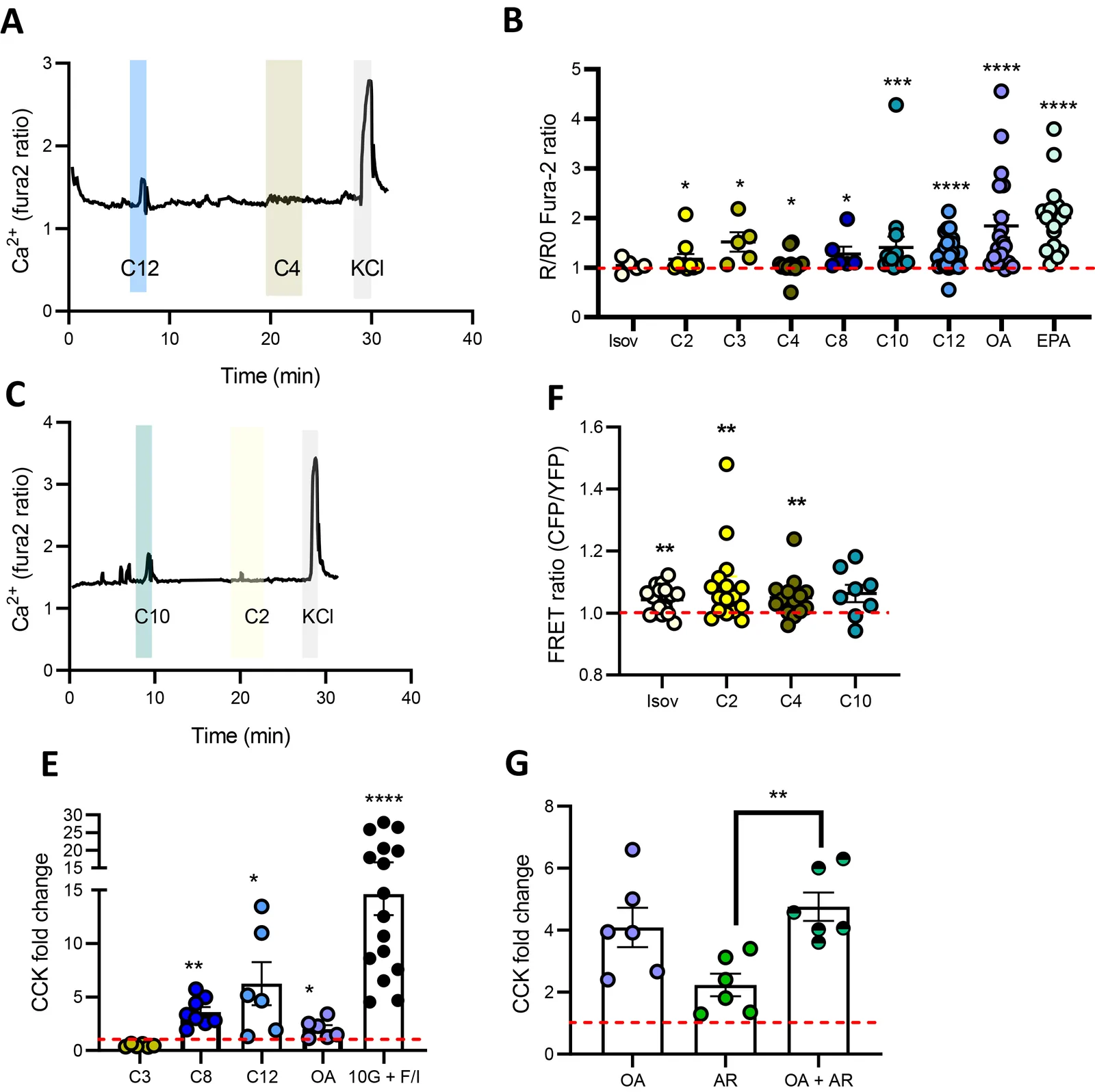
Stimulation of CCK release by different length chain fatty acids. (A, C) Representative Fura-2 (340/380) ratio traces of single I cells perfused with (A) dodecanoic acid (C_12_, 200 μM) and butyrate (C_4_, 1 mM), and (C) decanoic acid (C_10_, 1 mM) and acetate (C_2_, 1 mM). Both representative traces showed KCl as positive control (50 mM). (B) Data showing the fold change in R/R0 (R being the Fura2 ratio during perfusion of the stimulus and R0 being the Fura2 ratio during perfusion of basal solution) in response to Isovalerate (Isov, 1 mM), C_2_ (1 mM), propionate (C_3_, 1 mM), C_4_ (1 mM), octanoic (C_8_, 1 mM), C_10_ (1 mM), C_12_ (200 μM), oleic (OA, 200 μM), eicosapentanoic acid (EPA, 200 μM), (n = 10–19 cells from 3 to 6 independent experiments). (E, G) Secretion of CCK from CCK-Venus human duodenal organoids in response to the stimuli indicated, expressed as fold change vs basal condition (1 mM glucose and 0.01% bovine serum albumin (BSA)) measured in parallel. The stimuli included (E) C3 (1 mM), C8 (1 mM), C12 (200 μM) and OA (200 μM) and 10 mM glucose and 10 μM forskolin plus 100 μM IBMX as the positive control (10G + F/I). The stimuli included in (G) are OA (200 μM), AR (100 nM) and the combination OA + AR All test solutions contained 1 mM glucose and 0.01% BSA (n = 6 wells from 3 independent experiments). (F) Fold change of FRET ratio across different cells in response to Isov, C_2_, C_4_, and C_10_, all tested at 1 mM). Data are shown as ratio between maximal CFP/YFP ratio (R) during perfusion of stimulus and maximal CFP/YFP ratio (R0) during perfusion of basal solution (n = 10–19 cells from 3 to 6 independent experiments). Data are presented as mean ± SEM. Data are presented as mean ± SE. ∗p < 0.05, ∗∗p < 0.01, ∗∗∗p < 0.001, ∗∗∗∗p < 0.0001 b y one-sample Wilcoxon test (b, f, e) or by Kruskal–Wallis test (g).

**Figure 5:**
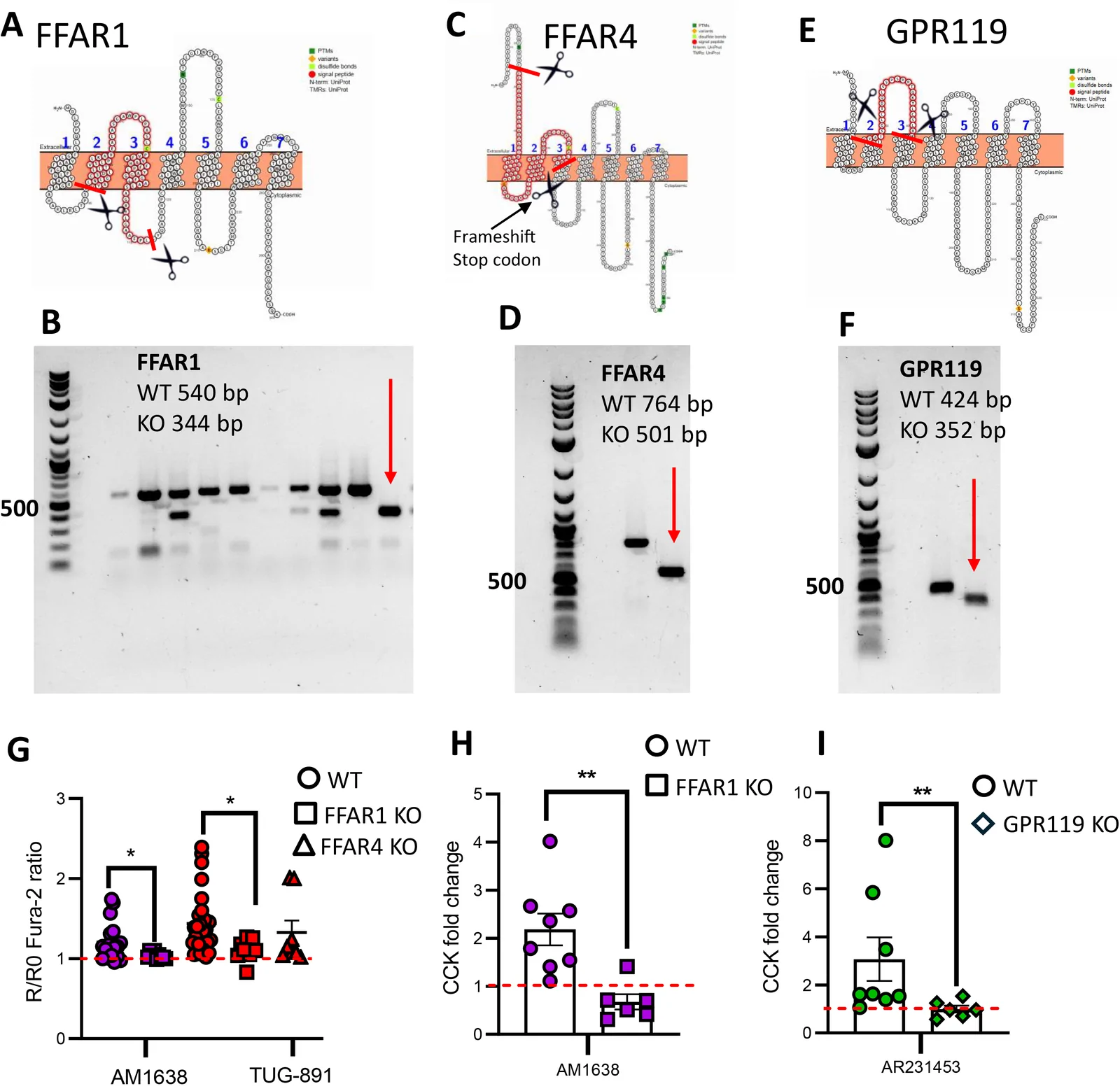
Generation and validation of *FFAR1*, *FFAR4*, and *GPR119* knock-out organoids. (A, C, E) Schematics representing FFAR1, FFAR4 and GPR119 CRISPR Cas9 KO strategy. Sequences between the scissors represent deleted regions. The topological structures of the two receptors were generated using Protter (version 1.0; https://wlab.ethz.ch/protter/start/). (B, D, F) Representative agarose gels showing PCR genotyping results for WT and FFAR1 (B), FFAR4 (D) and GPR119 (F) KO human CCK-Venus duodenal organoids. Expected band sizes for WT and KO are indicated in base pairs. (G) Increase in intracellular calcium levels in response to AM-1638 (10 μM) and TUG891 (30 and 1 μM) across I cells derived from WT (circles), FFAR1 KO (squares) and FFAR4 KO (triangles) organoids. The increase is shown as ratio between R (Fura-2 ratio during perfusion of stimulus) and R0 (Fura-2 ratio during perfusion of basal solution) (n = 4–11 cells from 3 to 4 independent experiments). (H, I) Secretion of CCK following stimulation with (H) AM-1638 (10 μM) in WT and FFAR1 KO organoids, and (I) AR231453 (100 nM) in WT GPR119 KO organoids. CCK release is expressed as fold change vs basal condition. All secretion experiments for KO lines were carried out in parallel with the WT line (n = 6 wells from 3 independent experiments). Data are presented as mean ± SEM. ∗p < 0.05, ∗∗p < 0.01 b y Mann–Whitney test (g, h, i).

**Figure 6:**
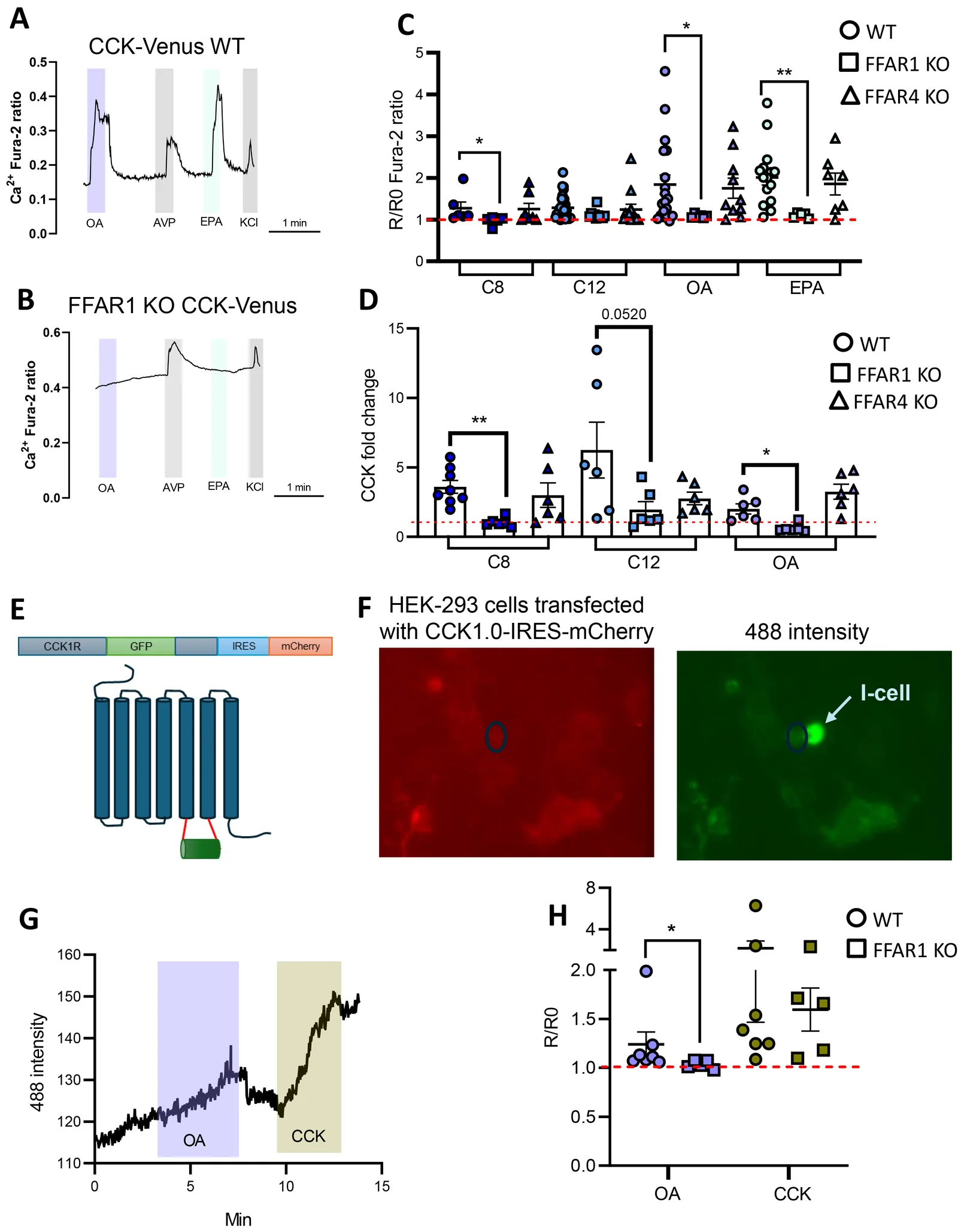
Unravelling the role of FFAR1, FFAR4 and GPR119 in fatty acid sensing in I cells. (A, B) Representative Fura-2 (340/380) ratio traces of single I cells perfused with oleic acid (OA, 200 μM) and eicosapentanoic acid (EPA, 200 μM) in (A) WT and (B) FFAR KO I-cells. Both representative traces showed KCl (30 mmol/l) and arginine vasopressin (AVP, 10 nmol/l) as positive control. (C) Increase in intracellular calcium levels in response to octanoic (C_8_, 1 mM), dodecanoic (C_12_, 200 μM), oleic acid (OA, 200 μM) and eicosapentanoic acid (EPA, 200 μM) across I cells derived from WT (circles), FFAR1 (squares) and FFAR4 (triangles) KO organoids. The increase is shown as ratio between R (Fura-2 ratio during perfusion of stimulus) and R0 (Fura-2 ratio during perfusion of basal solution) (n = 4–11 cells from 3 to 4 independent experiments). (D) Secretion of CCK following stimulation with C_8_ (1 mM), C_12_ (200 μM), and oleic acid (OA, 200 μM) in WT (circles), FFAR1 (squares) and FFAR4 (triangles) KO organoids. CCK release is expressed as fold change vs basal condition. All secretion experiments for KO lines were carried out in parallel with the WT line (n = 6 wells from 3 independent experiments). (E) Schematic showing CCK1 GRAB sensor plasmid construct. (F) Representative image of HEK-203 cells transfected with CCK1.0-IRES-mCherry construct and in co-culture with human I-cells from CCK-Venus duodenal organoids. (G) Representative Fura-2 (340/380) ratio trace of HEK293 transfected with CCK1R-IRES-mCherry Plasmid in co-culture with CCK-Venus duodenal organoids perfused with OA (200 μM) and CCK as positive control. (H) Data showing the fold change in R/R0 (R being the Fura2 ratio during perfusion of the stimulus and R0 being the Fura2 ratio during perfusion of basal solution) of the HEK293 transfected with CCK1R-IRES-mCherry plasmid in co-culture with WT or FFAR1 KO CCK-Venus duodenal organoids after perfusion with OA and CCK. (n = 5–6 cells from 3 different experiments). Data are presented as mean ± SEM. ∗p < 0.05, ∗∗p < 0.01 b y Mann–Whitney test and Kruskal–Wallis test (c, d, h). Statistical significance in (h) remained even after exclusion of the high value in the OA-WT group.

As low levels of the olfactory receptors OR51E1 and OR51E2 were detected, which are normally Gs coupled, intracellular cAMP levels in I-cells were monitored using the CCK-Epac-S-H187 line. The SCFAs C_2_, C_4_ and Isov (all at 1 mM) elicited modest but significant increases in cAMP levels whereas C_10_ did not elicit a significant increase in cAMP levels (Fig 4F). Significant responses to Isov were observed in 4/16 cells, whereas C_2_ and C_4_ induced CFP/YFP responses only in 2/16 and 1/15 CCK-Epac-S-H187 cells, respectively.

CCK secretion was measured in response to C_3_, C_8_, C_12_ and OA (Fig 4E). OA (200 μM) stimulated a significant increase in CCK secretion (2.0 ± 0.3-fold change), as did C_12_ (200 μM) and C_8_ (1 mM); 6.2 ± 2.0 and 3.6 ± 0.5-fold change, respectively (Fig 4E). OA was also tested in combination with the GPR119 agonist, resulting in a 4.7 ± 1.1-fold increase in CCK secretion, which was similar to that triggered by OA alone (Fig 4G). The SCFA C_3_ inhibited CCK release (0.5 ± 0.06 fold change) (Fig 4E), suggesting that the Gi component of FFAR2 signalling, and possible FFAR3, expression of which we observed previously in the I-cell subcluster of Chromogranin A labelled human duodenal organoids [18], but initially failed to detect here, dominates over the observed small Gs coupled olfactory receptor responses. We revisited the RNAseq data of the FACsorted I-cells including multimapped reads, which are normally filtered out, given that *GPR42*, a gene with 98–99% sequence identity with *FFAR3* [35], could potentially affect *FFAR3* read identification; in this analysis we did find an enrichment of *FFAR3* expression in Venus labeled I-cells (Supplementary Fig. 1) suggesting that this receptor contributes to the overall inhibitory effect of propionate on secretion. Additionally, we aimed to evaluate potential synergistic effects between FFAR1 and GPR119 activation through their respective elevation of intracellular Ca^2+^ and cAMP levels. To this end, we tested OA and AR231452 both individually and in combination to assess CCK secretion in human organoids. The combination elicited a 4.8 ± 0.5-fold increase in CCK secretion, whereas OA and AR231452 (AR) alone induced 4.1 ± 0.6-fold and 2.2 ± 0.4-fold increases, respectively, respectively, not supporting a synergistic effect under these experimental conditions (Fig 4G).

### 3.5 Unravelling the relative importance of FFAR1, FFAR4 and GPR119 in lipid sensing in I-cells

Two pairs of sgRNAs were used to delete transmembrane domains 2 and 3 of FFAR1, or extracellular loop 2 of GPR119, following the same strategy as previously published (Figure 5A,E) [25]. To knock-out FFAR4, two sgRNAs (Table S1) were used to delete transmembrane domain 1 and 2 (Fig 5C). Successful biallelic KO of the receptors was confirmed by PCR screening of surviving CCK-Venus organoids post gene editing of FFAR1 (Fig 5B), GPR119 (Fig 5F) and FFAR4 (Fig 5D). Successful KO of FFAR4 was validated by Sanger sequencing, confirming disruption of the downstream coding region by introduction of a frameshift (Fig. S1).

FFAR1 KO CCK-Venus I-cells showed a significant impairment of intracellular Ca^2+^ responses to the specific agonist AM1638 (10 μM) and decreased responses to the FFAR1/4 agonist TUG-891 (30 μM) compared with WT cells (Fig 5G). By contrast, intracellular Ca^2+^ responses to TUG-891 (30 μM) were not significantly impaired in FFAR4 KO I-cells (Fig 5G). Consistent with these findings, CCK release in response to AM-1638 was abolished in FFAR1 KO organoids (0.7 ± 0.2 compared with a 2.2 ± 0.3 fold change in WT-organoids in parallel secretion experiments) (Fig 5H). Secretion studies also confirmed functional deletion of GPR119, as evidenced by the impaired secretory response to the specific agonist AR231453 (1.0 ± 0.1 fold change) compared with WT I-cells (3.07 ± 0.9 fold change) (Fig 5I).

Having validated the receptor KOs, we tested their responses to different fatty acids. Ca^2+^ responses were evaluated in I-cells from FFAR1 and FFAR4 KO organoids perfused with C_8_, C_12_, OA and EPA (1 mM for C_8_, 200 μM for C_12_, OA and EPA) (Figure 6A,B, c). Responses to C_8_, OA and EPA were not significantly different from WT in I-cells from FFAR4 KO organoids, but were blunted in I-cells from FFAR1 KO organoids (Fig 6C). Ca^2+^ responses to C_12_ were not statistical different between WT, FFAR1 and FFAR4 KO I-cells, but this may reflect the high number of non-responding I-cells from the WT organoids (10/29 non-responders) (Fig 6C). CCK release was measured in response to C_8_, C_12_ and OA (Fig 6D). CCK secretion levels were similar in cells from WT and FFAR4 KO organoids in response to C_8_ (1 mM, 3.6 ± 0.5 and 3.0 ± 0.9 fold change, respectively), and OA (200 μM, 2.0 ± 0.3 and 3.2 ± 0.5 fold change, respectively) (Fig 6D). By contrast, secretory responses to C_8_ and OA were significantly blunted in cells from FFAR1 KO (1.0 ± 0.1 fold and 0.6 ± 0.1 fold changes, respectively) (Fig 6D). The secretory response to C_12_ (200 μM) showed a non-significant trend towards impairment in FFAR1 KO cells (6.2 ± 2.0 fold in WT vs 2.0 ± 0.6 in FFAR1 KO, and 2.8 ± 0.5 fold change in FFAR4 KO).

We also monitored CCK release from single I-cells in real time using a GPCR-activation-based (GRAB) sensor (Fig 6E). The CCK1.0-IRES-mCherry GRAB biosensor [27] was transfected into HEK293T cells, which were then co-cultured with digested CCK–Venus duodenal organoids (Fig. 6F). This setup allowed us to monitor CCK release using biosensor-expressing HEK cells (identified by their mCherry fluorescence) surrounding identified I-cells (Venus positive, not red). At the end of each experiment, an effective concentration of CCK was applied to fully activate the GRAB-CCK1.0 sensor (Fig. 6H). In experiments using WT CCK-Venus organoid cells, perfusion with OA (200 μM) triggered increases in the fluorescence intensity of biosensor HEK cells in the vicinity of I-cells, indicating local release of CCK (Figure 6G,H). The same experiment was performed using FFAR1 KO CCK-Venus duodenal organoids, where there was no increase in the GRAB-CCK1.0 fluorescence intensity when perfusing with OA (Fig 6H). These results are consistent with the I-cell Ca^2+^ imaging results and supernatant CCK measurements described earlier (Figure 6C,D).

## 4 Discussion

The molecular physiology of I-cells has previously been demonstrated in mouse organoids, cell lines and primary intestinal cultures [15,17,36]. However, characterisation of I-cells in human *in vitro* models remains limited, due to difficulties in identifying I-cells and in reliably detecting CCK-secretion. In this study, we established a human duodenal organoid model with fluorescently labelled I-cells by inserting a fluorescent reporter gene at the 3′ end of the CCK gene using CRISPR-Cas9 technology. This strategy enabled the identification and isolation of CCK-secreting cells for transcriptomic, peptidomic, and functional analyses, including secretion assays, electrophysiology, and live-cell imaging of Ca^2+^ and cAMP dynamics. In combination with a targeted LC-MS/MS-based assay [31], we demonstrate that I-cell detection of LCFAs and MCFAs is predominantly FFAR1-dependent, with little contribution of FFAR4. The development of the CCK-Venus and CCK-Epac-S-H187 reporter lines represents a significant advance, enabling detailed characterisation of the cellular and molecular properties of human I-cells *in vitro*.

Transcriptomic and peptidomic profiling of human I-cells revealed the expression and translation of a broad range of peptide hormones in addition to CCK, including gastrin, PYY, GIP, NTS, GCG and MLN. UCN3 mRNA was detected at moderately high levels in I-cells although could not be confirmed at the protein level with the LC-MS/MS protocol used here. The large overlap of I-cells with other enteroendocrine cell types in the upper small intestine is consistent with data from mouse models, such as CCK-GFP [37] and CCK-tdTomato [16]; and human organoid systems with neurogenin-3 (NEUROG3) overexpression or chromogranin-A (CHGA)-Venus reporters [18,38]. Supporting these observations, immunohistochemical analyses of human jejunum have shown colocalisation of CCK with GLP-1, PYY, GIP, and SCT [39]. Bulk RNA-seq analysis further defined the ion channel expression profile of human I-cells, revealing conserved features across enteroendocrine subtypes [21,24]. Previous electrophysiological studies were performed in isolated mouse CCK-GFP cells [40], but the present study provides the first direct evidence that human I-cells generate action potentials in response to membrane depolarisation.

Bulk RNA-seq further revealed enriched expression of a range of lipid-related GPCRs in human I-cells, including *FFAR1*, *FFAR2*, *GPR119* and *GPBAR1*, consistent with previous studies in mouse [16,17]. The results mirror our previous single-cell RNAseq analysis of human duodenal EECs from CHGA-Venus organoids, but with the greater read-depth provided by bulk RNA sequencing [18] The previous single cell analysis offers additional information about I-cell heterogeneity but is less sensitive for detecting low-abundance GPCRs. Nevertheless, the comparable findings between these datasets support the validity of the CCK-Venus reporter line and demonstrate the reproducibility of the receptor expression profile across independent transcriptomic datasets. Importantly, the current work extends these findings by establishing a functional link between receptor expression, intracellular signalling, and CCK secretion.

Functionally, we show that human I-cells respond to a variety of nutrients and small molecules, leading to elevations in intracellular second messengers (Ca^2+^ and cAMP) and subsequent CCK secretion. Using CRISPR-Cas9-mediated gene knockout, we further investigated the roles of individual receptors in regulating CCK secretion. The results highlighted the key importance of FFAR1 for fatty acid sensing in I-cells. The role of I-cell FFAR1 for detecting oleate was further confirmed using a GPCR-activation-based (GRAB) sensor, which allows monitoring of local CCK secretion from single I-cells in real time. These findings align with previous mouse studies showing that *Ffar1* KO significantly reduced plasma CCK levels following gastric gavage of olive oil [19] or corn oil [20]. Linolenic acid–induced Ca^2+^ responses and CCK secretion in I cells isolated from Cck-GFP mice were also abolished by *Ffar1* KO, supporting a central role for FFAR1 in long-chain fatty acid sensing [19]. FFAR4 was highly expressed in both Venus-positive and -negative populations, and is commonly studied using the agonist TUG-891. A previous study indicated that TUG-891 is selective for FFAR4 at low concentrations (0.01–1 μM), while at higher concentrations it can also activate FFAR1 [32]. Here we showed that only a high TUG-891 concentration (30 μM) elicited a detectable Ca^2+^ response, suggesting this is an off-target effect on FFAR1. Although FFAR4 is commonly described as Gq/11- and β-arrestin–coupled, it has also been reported to signal via Gi/o proteins in a ligand- and cell type–dependent manner, leading to inhibition of cAMP production [33]. Supporting this, FFAR4 activation has been shown to regulate the release of satiety hormones via pertussis toxin–sensitive Gi proteins [41]. In human I-cells, we found no evidence of FFAR4 coupling to Gq-mediated Ca^2+^ signalling, but it is possible it could instead engage Gi pathways.

Human FFAR4 exists as two spliced isoforms, with the long variant containing a 16–amino acid insertion in intracellular loop 3 [33]. These authors reported that the long variant exhibits relatively weak Gi activity and no detectable Gs or Gq signalling in response to EPA, TUG-891, or LA, while retaining the ability to recruit β-arrestins [33]. By PCR, we found that human duodenal organoids express the long *FFAR4* transcript (Fig. S2), which may explain the lack of intracellular calcium response to TUG-891 at 1 μM. Future studies employing BRET-based biosensors could provide additional mechanistic insight into whether FFAR4 couples to non-canonical intracellular signalling pathways in I-cells.

CCK secretion and cAMP levels in human I-cells were modestly stimulated by GPR119 agonism, suggesting a role for GPR119 in monoacylglycerol-driven CCK release in humans. These results are consistent with human studies showing that ingestion of 1,3-di-butyryl-2-oleoyl glycerol (C4 dietary oil) elevated plasma CCK levels, although to a lesser extent than olive oil [12]. By contrast, oral administration of the structurally related 1,3-dioctanoyl-2-oleoyl glycerol (C8 dietary oil) surprisingly did not appear to stimulate CCK release [42]. The authors speculated that faster hydrolysis of the C4 moieties compared to C8 moieties might allow sufficient time for conversion of the product 2-mono-oleoyl-glycerol to 1-mono-oleoyl-glycerol and subsequent oleate release to stimulate free fatty acid receptors. Whilst this seems plausible, our observation of a secretory response to C_8_ suggests that maybe slower digestion of C_8_ dietary oil prevents sufficient production of either GPR119 or FFAR1 agonists in the proximal duodenum, where CCK-expression is highest. Others have recently reported enhanced (compared to FFAR1 agonism alone) CCK-secretion in humans when FFAR1 and GPR119 agonist were combined, although this seemed not statistically significant [43], consistent with our failure to observe a clear synergistic effect when co-stimulating these receptors. It seems likely that due to lipid-digestion kinetics FFAR1 is of greater importance in the proximal small intestine, where most I-cells are located.

The bile acid receptor GPBAR1 is highly enriched in human I-cells, but there is limited direct evidence that GPBAR1 acts as a regulator of CCK release *in vivo*. In this study we showed that GPBAR1 agonism increased both intracellular cAMP levels and CCK secretion in human organoids, suggesting a potential role for bile acid signalling in I-cells after lipid ingestion. The contribution of bile acids to physiological CCK regulation is not fully understood. Bile acids may influence CCK secretion indirectly by promoting lipid digestion, thereby increasing the availability of fatty acids and monoacylglycerols that trigger CCK release. GPBAR-A has been identified as a strong stimulus of other enteroendocrine hormones, including GIP, GLP-1 and PYY [44,45].

A range of fatty acid chain lengths (C_2_–C_4_, C_8_, C_10_, C_12_, OA and EPA) increased intracellular Ca^2+^ levels in human I-cells, consistent with activation of FFAR1 and FFAR2, the latter being Gi or Gq coupled. Isovalerate, C_2_ and C_4_ modestly increased cAMP, compatible with activation of Gs-coupled olfactory receptors, of which we detected both OR51E1 and OR51E2, with the latter but not the former being more highly expressed in I-cells than their neighbours. These olfactory receptors (mouse homologues Olfr558 and Olfr78) respond to short- and medium-chain carboxylic acids [46,47] and have been identified in various EECs, including L-cells [24,48] and enterochromaffin cells [28,49] although their role in hormone secretion remains poorly defined.

Medium- and long-chain fatty acids such as C_8_, C_12_, and oleic acid significantly stimulated CCK release in human duodenal organoids, whereas the SCFA C_3_ was, if anything, inhibitory, consistent with *in vivo* findings in pigs where intra-cecal administration of propionate suppressed CCK secretion [50] and human studies, where consumption of tributyrin did not increase plasma CCK [42]. Fatty acid chain length dependent CCK release has been reported previously *in vitro* [51] and *in vivo* [10]. In humans, intragastric and intraduodenal infusion of C_12_ increased plasma CCK concentrations more effectively than C_10_ [10,11]. Supporting this, *in vitro* studies demonstrate a clear chain length dependence, with C_6_ and C_8_ failing to stimulate CCK secretion in murine STC-1 cells, C_10_ only effective at high concentrations (>500 μM), and C_12_ inducing secretion at lower concentrations (∼100 μM). In our study, C_8_, C_12_, and oleic acid all triggered CCK release in 2D duodenal organoids, consistent with previous findings of elevated plasma CCK after orogastric administration of C_8_, C_12_ and OA [52].

Previous studies have reported that Ffar1 and Gpr119 agonists acted synergistically to enhance GLP-1 release [53]. However, the role of GPR119 in human CCK secretion remains unclear. In our study, the combination of OA with the GPR119 agonist AR231453 produced an additive, but not synergistic, increase in CCK secretion, with OA being the stronger stimulus. Supporting these results, a recent study found increased CCK levels in humans over a 24 h period on treatment with the FFAR1 agonist K-757, but this was not further increased by co-treatment with the GPR119 agonist K-833. By contrast, the combined GPR119 and FFAR1 treatment resulted in greater GLP-1 and PYY secretion compared with either treatment alone [43]. These combined data highlight the critical role of FFAR1 in FA-induced CCK secretion in human I-cells, while the contribution of GPR119 appears limited and context-dependent.

## 5 Conclusions

This study identified several nutrient-sensing GPCRs and transporters enriched in human I-cells, providing important insight into the molecular mechanisms governing CCK release. The results support the critical role for FFAR1 in CCK secretion triggered by lipid ingestion, with lesser roles for GPBAR1 and GPR119, and little evidence for a role for FFAR4. The results significantly advance our understanding of the molecular mechanisms of how lipids trigger CCK release and establish a foundation for the development of targeted, physiology-driven therapies for obesity and metabolic disease.

## CRediT authorship contribution statement

**Marta Santos-Hernández:** Writing – review & editing, Writing – original draft, Visualization, Methodology, Funding acquisition, Formal analysis, Data curation, Conceptualization. **Mae Tabbada:** Writing – review & editing, Investigation, Formal analysis, Data curation. **Nademah Tamkin:** Writing – review & editing, Investigation, Formal analysis, Data curation. **Linxia Li:** Writing – review & editing, Investigation, Formal analysis, Data curation. **Nunzio Guccio:** Writing – review & editing, Investigation, Formal analysis, Data curation. **Adam Davison:** Writing – review & editing, Investigation, Formal analysis, Data curation. **Chris A. Smith:** Writing – review & editing, Investigation, Formal analysis, Data curation. **Richard Kay:** Writing – review & editing, Investigation, Formal analysis, Data curation. **Constanza Alcaino:** Writing – review & editing, Investigation, Formal analysis, Data curation. **Mirza Baig:** Writing – review & editing, Investigation, Formal analysis, Data curation. **Austin Punnoose:** Writing – review & editing, Investigation, Formal analysis, Data curation. **Yining Xu:** Writing – review & editing, Investigation. **Rula Bany Bakar:** Writing – review & editing, Investigation. **Frank Reimann:** Writing – review & editing, Writing – original draft, Supervision, Project administration, Funding acquisition, Conceptualization. **Fiona M. Gribble:** Writing – review & editing, Writing – original draft, Supervision, Project administration, Funding acquisition, Conceptualization.

## Ethics declaration

Written informed consent to take part in the study and to publish the article has been obtained from all participants or their legal representatives. The privacy rights of participants have been observed.

This study included organ or tissue donors. This study includes human biological material and consent was obtained by donors, or their next of kin or legal representatives, for use in this study and for publication of the article. The samples used in this research were not sourced from executed prisoners or prisoners of conscience.

This study was performed in compliance with relevant laws, regulatory frameworks and guidelines where the research took place. This study was approved by the East of England–Cambridge Central Research Ethics Committee. (Approval No. 09/H0308/24)

## Funding

This research was funded by a 10.13039/100004440Wellcome joint investigator award to 10.13039/100031945FR/FMG (220271/Z/20/Z) and the MRC-Metabolic Diseases Unit program 3 to FR/FMG (MRC_MC_UU_12,012/3). M.S–H was awarded with the Martín Escudero postdoctoral Fellowship, followed by a Marie Sklodowska-Curie Postdoctoral Fellowship adopted and funded by 10.13039/100014013UK Research and Innovation EP/Y036824/1. Core support was provided by the 10.13039/501100000265MRC (MRC_MC_ UU_00014/5) and 10.13039/100004440Wellcome (100574/Z/12/Z). The LC-MS/MS instrument was funded by the 10.13039/501100000265MRC (MR/M009041/1).

## Declaration of competing interest

The authors declare the following financial interests/personal relationships which may be considered as potential competing interests:Prof. Frank Reimann reports was provided by Frank Reimann. Frank Reimann reports a relationship with AstraZeneca and Glaxo-Smith-Clime and Eli Lilly & Company that includes: funding grants. Frank Reimann has given an invited research talk at Boehringer Ingelheim in 2025 - this did eventually not result in any formal collaboration, as the University and Boehringer Ingelheim were unable to agree terms If there are other authors, they declare that they have no known competing financial interests or personal relationships that could have appeared to influence the work reported in this paper.

Fiona M Gribble reports financial support was provided by Antag Therapeutics ApS. Fiona M Gribble reports a relationship with Antag Therapeutics ApS that includes: consulting or advisory. If there are other authors, they declare that they have no known competing financial interests or personal relationships that could have appeared to influence the work reported in this paper.

## Data Availability

Data will be made available on request.
