# Supplementary material for: Molecular mechanisms of lipid sensing in human cholecystokinin-secreting enteroendocrine cells
Source: Mol Metab. 2026 Sep 28;114:102448. doi: 10.1016/j.molmet.2026.102448 (PMC13677200; doi:10.1016/j.molmet.2026.102448)
Supplement: Supplementary file 1 [file mmc1.docx]

**Supplemental Material for: “Molecular mechanisms of lipid sensing in human cholecystokinin-secreting enteroendocrine cells”**

| **Targeting gene** | **Strand** | **gRNA sequence** | **PAM** |  | **Oligos** |
| --- | --- | --- | --- | --- | --- |
| CCK | - | GCGGCTGGGTCCTCTAGGAG | GGG | FW | CACCGCGGCTGGGTCCTCTAGGAG |
|  |  |  |  | RV | AAACCTCCTAGAGGACCCAGCCGC |
| FFAR1 | + | CTGGTCTACGCCCTGAACCT | GGG | FW | caccgCTGGTCTACGCCCTGAACCT |
|  |  |  |  | RV | aaacAGGTTCAGGGCGTAGACCAGc |
|  | - | CGGAAGGCTTGGTAGCCCAA | GGG | FW | caccgCGGAAGGCTTGGTAGCCCAA |
|  |  |  |  | RV | aaacTTGGGCTACCAAGCCTTCCGc |
| FFAR4 | + | CCCTTCTTCTCCGACGTCAA | GGG | FW | caccgCCCTTCTTCTCCGACGTCAA |
|  |  |  |  | RV | aaacTTGACGTCGGAGAAGAAGGGc |
|  | + | GGGTCATCACGTAGAAGAGC | AGG | FW | caccGGGTCATCACGTAGAAGAGC |
|  |  |  |  | RV | aaacGCTCTTCTACGTGATGACCC |
| GPR119 | + | AGAGATGGCCACACCAATCA | AGG | FW | caccgAGAGATGGCCACACCAATCA |
|  |  |  |  | RV | aaacTGATTGGTGTGGCCATCTCTc |
|  | + | AAGTGACAAATGCCATCCGC | AGG | FW | caccgAAGTGACAAATGCCATCCGC |
|  |  |  |  | RV | aaacGCGGATGGCATTTGTCACTTc |

**Table S1.** The sequence of oligos used in synthesis of sgRNA plasmid for human duodenal knock-in (CCK-Venus and CCK-Epac-S-H187) and knock-out (FFAR1, FFAR4 and GPR119 KOs).

Cholecystokinin (CCK), free fatty acid receptor 1 (FFAR1), free fatty acid receptor 4 (FFAR4), monoacyl-glycerol receptor GPR119, forward (FW), reverse (RV)

**Table S2.** Table S1. PCR primers used for the construction of the CCK-Venus and CCK-Epac-S-H187 plasmids and for genotyping of the generated knock-in and knock-out (KO) human duodenal organoids.

| **Description** |  | **Oligos** |
| --- | --- | --- |
| End of CCK LHA + start of P2A | fw | AGGAGTATGAGTACCCCTCCGGAAGCGGAGCTACTAACTT |
| Start of CCK RHA + end of fxNeo | rv | GATGGCGGCTGGGTCCTCTATAGTGGATCCCCTCGAGGGA |
| End of CCK RHA + start TOPO backbone | fw | TGGACAGTGAAAGGGCCATCAAGGGCGAATTCTGCAGATA |
| Start of CCK LHA + end of TOPO backbone | rv | CCTGACTCCTGGAGAGCTCTCGAATTCCAGCACACTGGCG |
| End of TOPO backbone + start CCK LHA | fw | CGCCAGTGTGCTGGAATTCGAGAGCTCTCCAGGAGTCAGG |
| Start of P2A + end of CCK LHA | rv | AAGTTAGTAGCTCCGCTTCCGGAGGGGTACTCATACTCCT |
| End of fxNeo + start of CCK RHA | fw | TCCCTCGAGGGGATCCACTATAGAGGACCCAGCCGCCATC |
| Start of TOPO backbone + end CCKRHA | rv | TATCTGCAGAATTCGCCCTTGATGGCCCTTTCACTGTCCA |
| End of P2A + start of mTurq (for CCK-Epac-S-H187) | fw | TGGAGGAGAACCCTGGACCTATGGCGGCCGCAATGGTG |
| Start of mTurq + end of P2A (for CCK-Epac-S-H187) | rv | CACCATTGCGGCCGCCATAGGTCCAGGGTTCTCCTCCA |
| CCK outside LHA for screening 5' integration | fw | TCACAGCAGGCAGAAGGATG |
| CCK outside RHA for screening 3' integration | rv | TTAAAGGCCAAGAGAACCCCA |
| start of Venus; use for 5' integration CCK-Venus | rv | GGACACGCTGAACTTGTGG |
| Fw in fx Neo; use for 3' integration CCK-Venus and CCK-Epac | fw | TGGCTACCCGTGATATTGCT |
| rv in Epac1; use for 3' integration CCK-Epac | rv | GCTGCCATCACCTCTCTCACG |
| FFAR4 KO screening | fw | TCAGTCGCCTCCCAGATGAG |
| FFAR4 KO screening | rv | GGCACAAGATTTGGCACAAC |
| FFAR1 KO screening | fw | AGCCAGGTTGCACACAGGAC |
| FFAR1 KO screening | rv | GTTGACCGGTGTGTTGATGC |
| GPR119 KO screening | fw | AGTGAAGTCCTGCCACTTCG |
| GPR119 KO screening | rv | AAGACACTAACCACAGCCCG |

**Fig S1**

**A. FFAR4**

**B. FFAR4 Knock-out**

Fig S1. Sequence analysis of the human FFAR4 locus generated using SnapGene Viewer. (a) Fragment of the FFAR4 gene sequence in CCK-Venus WT organoids. (b) Corresponding fragment of the FFAR4 gene sequence in FFAR4 KO organoids. The highlighted region indicates the mutation introduced by genome editing, confirming disruption of the downstream coding sequence through the generation of a frameshift.

**Fig S2**

Fig S2. Representative agarose gels showing PCR products amplified from cDNA generated from RNA isolated from CCK-Venus organoids. Expected band sizes are indicated in base pairs (bp).
